# Effects of dietary components on high-density lipoprotein measures in a cohort of 1,566 participants

**DOI:** 10.1186/1743-7075-11-44

**Published:** 2014-09-15

**Authors:** Daniel Seung Kim, Amber A Burt, Jane E Ranchalis, Leah E Jarvik, Jason F Eintracht, Clement E Furlong, Gail P Jarvik

**Affiliations:** Department of Medicine, Division of Medical Genetics, University of Washington School of Medicine, Box 357720, Seattle, WA 98195-7720 USA; Department of Genome Sciences, University of Washington School of Medicine, Seattle, WA USA; Department of General Medicine, Virginia Mason Medical Center, Seattle, WA USA

**Keywords:** HDL, HDL-C, HDL-2, HDL-3, Apolipoprotein A1, HDL subfractions, Folate, Alcohol, Fatty acids, Magnesium, Food frequency questionnaire, Cardiovascular disease

## Abstract

**Background:**

Recent data suggest that an increased level of high-density lipoprotein cholesterol (HDL-C) is not causally protective against heart disease, shifting focus to other sub-phenotypes of HDL. Prior work on the effects of dietary intakes has focused largely on HDL-C. The goal of this study was to identify the dietary intakes that affect HDL-related measures: HDL-C, HDL-2, HDL-3, and apoA1 using data from a carotid artery disease case–control cohort.

**Methods:**

A subset of 1,566 participants with extensive lipid phenotype data completed the Harvard Standardized Food Frequency Questionnaire to determine their daily micronutrient intake over the past year. Stepwise linear regression was used to separately evaluate the effects of dietary covariates on adjusted levels of HDL-C, HDL-2, HDL-3, and apoA1.

**Results:**

Dietary folate intake was positively associated with HDL-C (p = 0.007), HDL-2 (p = 0.0011), HDL-3 (p = 0.0022), and apoA1 (p = 0.001). Alcohol intake and myristic acid (14:0), a saturated fat, were each significantly associated with increased levels of all HDL-related measures studied. Dietary carbohydrate and iron intake were significantly associated with decreased levels of all HDL-related measures. Magnesium intake was positively associated with HDL-C, HDL-2, and HDL-3 levels, but not apoA1 levels, while vitamin C was only associated with apoA1 levels. Dietary fiber and protein intake were both associated with HDL-3 levels alone.

**Conclusions:**

This study is the first to report that dietary folate intake is associated with HDL-C, HDL-2, HDL-3, and apoA1 levels in humans. We further identify numerous dietary intake associations with apoA1, HDL-2, and HDL-3 levels. Given the shifting focus away from HDL-C, these data will prove valuable for future epidemiologic investigation of the role of diet and multiple HDL phenotypes in heart disease.

## Background

The strong inverse association between measures of high density lipoprotein cholesterol (HDL-C) and cardiovascular disease risk [1] has recently prompted several studies to establish the role of HDL-C in the causal pathway of atherosclerosis and its resulting end-organ damage. However, in both clinical trials [2, 3] and Mendelian randomization analyses [4], increasing HDL-C levels has failed to demonstrate a significant decrease in cardiovascular disease, raising doubts as to the cardioprotective nature of the HDL-C and the high density lipoprotein particle (HDL-P) in its entirety.

However, recent evidence from the Multi Ethnic Study of Atherosclerosis (MESA) suggests that aspects of the HDL-P not measured by HDL-C may be responsible for the cardioprotective effects of HDL [5]. In this work, Mackey *et al*. studied a cohort of 5,598 participants, measured both HDL-P (which reflects the total quantity of HDL and its associated proteins) and HDL-C, and performed multivariate regression on the outcomes of incident coronary heart disease (CHD) and carotid intima media thickening (cIMT). From these analyses, Mackey *et al*. found that when HDL-P was already included in the model, HDL-C measures no longer were protective against cardiovascular disease risk. This finding suggested that there were elements of HDL likely responsible for its cardioprotective nature that were better reflected by HDL-P than HDL-C.

HDL is broadly composed of two sub-species, HDL-2 and HDL-3, each of which have distinct biochemical, physiologic, and metabolic functions [6]. HDL-2 has a much higher density of apolipoprotein A1 (apoA1), whose levels have been consistently associated with cardioprotection [7, 8]. HDL-3 is the smaller, denser, and more lipid-poor of the two sub-fractions of HDL. However, HDL-3 is strongly antioxidant [9] and also is closely associated with the glycoprotein enzyme, paraoxonase-1 (PON1) [10]. PON1 is itself atheroprotective [11–14] and can prevent LDL [15, 16] and HDL oxidation [17] (other functions of PON1 are summarized in a recent review article [18]).

We have previously determined that HDL-3 was a superior predictor of carotid artery disease (CAAD) compared with HDL-C, HDL-2 or apoA1, considering 1,725 participants in a CAAD case–control cohort [19]. When HDL-3 was included in the model, none of the other measures of HDL were significantly associated with CAAD [19]. Moreover, the CAAD-protective effects of HDL-3 were independent of its closely associated enzyme, (PON1) [9] which is itself inversely associated with CAAD [14, 20, 21], suggesting that unmeasured elements of HDL-3 may be responsible for cardioprotective effects in our data [6].

Diet is one of the key behavioral targets for preventing cardiovascular disease [22, 23]. Specific diets, such as the Mediterranean [24] and DASH [25] diets have been associated with lower incidence of CHD. These favorable results are largely attributed to improved biomarker profiles, including increases in HDL-C [26]. However, studies have not generally analyzed the effects of diet on the more specific measures of HDL [24–26], such as HDL-2, HDL-3, and apoA1, all of which may be more closely associated with the cardioprotective elements of HDL [19].

We previously have used food frequency questionnaire data in our data to identify novel dietary micronutrient intakes that affect PON1 enzyme activity [27–29]. In this work we have leveraged our large and well-characterized Carotid Lesion Epidemiology And Risk (CLEAR) study in conjunction with thorough dietary intake data to determine the micronutrient determinants of each specific measure of HDL. Specifically, we sought to determine what specific dietary micronutrients are associated with HDL-C, HDL-2, HDL-3, and apoA1, and how these dietary associations differ across the various measures of HDL.

## Methods

### Ethics statement

Institutional review boards at the University of Washington, Virginia Mason Medical Center, and Veterans Affairs Puget Sound approved the CLEAR study. Written, informed consent was obtained from each participant of the study.

### Sample

The CLEAR study is a Seattle-based prevalent CAAD case–control study, composed primarily of veterans, with controls matched by age at diagnosis (for CAAD cases) and current unaffected age (for controls). Exclusion criteria included familial hypercholesterolemia, total fasting cholesterol greater than 400 mg/dl, hypocoagulable state and/or the use of anticoagulant medication, post-organ transplant, or the inability to consent. The study population for this analysis was a subset (n = 1,566) of the previously described CLEAR study [14, 20] with both dietary intake and HDL data. All participants in the studied subset presented had complete covariate data. The studied subset consisted of 433 participants with CAAD as determined by ultrasound (>50% stenosis in either carotid artery), 70 participants with moderate obstruction (15-49% obstruction in at least one carotid artery), 3 subjects with other phenotypes, including peripheral artery disease (PAD) and coronary artery disease (CHD), and 1060 controls (<15% carotid stenosis bilaterally and absence of PAD and CHD). Of the 1,566 outpatients enrolled in this subset of the CLEAR study, 60% were recruited from Veterans Affairs Puget Sound, 22% from the University of Washington, and 18% from Virginia Mason Medical Center. Current smoking status and reported ancestry were obtained by self-report. For the purposes of our analyses, diabetes was defined by hemoglobin A1C ≥ 6.5% and/or hypoglycemic medication or insulin use, which was determined via self-report matched to hospital pharmacy records.

### Lipid measurements

Standard methods were used to determine total cholesterol, triglycerides, and HDL in fasting whole plasma using an Abbott Spectrum analyzer. HDL fractions 2 and 3 were determined by precipitating HDL-2 from isolated total HDL, measuring HDL-3 in the supernatant, and subtracting this from total HDL to obtain HDL-2 [30]. Apolipoprotein A-I was measured as previously described [31]. All lipid measurements had approximate standard distributions.

### Food-frequency questionnaire

At enrollment, participants were asked to complete the standardized Harvard food frequency questionnaire (FFQ) developed by the Health Professionals Follow-Up Study (https://regepi.bwh.harvard.edu/health/nutrition.html). The FFQ asked about i) the average frequency of intake over the previous year of specified portions of 131 foods and ii) the use of vitamins and mineral supplements, including the dose and duration of use. Questions regarding brand of multivitamins and cereal used were asked to clarify the quantities of specific vitamin supplementation. All vitamin usage was energy-adjusted to 2,000 kcal/day. The surveys were then returned to Harvard School of Public Health and the Brigham and Women’s Hospital, where they underwent quantitative analysis to return the inferred average intake of 162 specific and unique dietary nutrient intakes. Of the 162 returned dietary nutrient intakes, 53 non-redundant intakes with complete data across all participants with FFQ diet and plasma lipid data were carried forward to analysis. The Harvard Food Frequency Questionnaire has been validated against two, in-depth, 1-week diet records taken approximately six months apart [32]. Additionally, the inferred intake of dietary fatty acids and cholesterol have been validated against plasma lipid measurements [33, 34].

### Statistical methods

Natural log transformation was performed for each of the 53 specific dietary micronutrient intake variables. Extreme observations were Winsorized [35] to 3 standard deviations from the mean before natural log transformation. For food frequency data, participants were excluded if their caloric intake was <800 calories/day or >4000 calories/day. Additionally, participants were excluded if the returned survey had ≥70 missing items.

We performed separate stepwise linear regression models on the individual lipid phenotypes of apo-A1, HDL-C, HDL-2, and HDL-3, with all 53 natural-log transformed dietary intakes included in the model. Model comparison was performed using Akaike’s information criterion (AIC), beginning with a base model comprised of age, sex, diabetes status, current smoking, and dummy variables for self-reported African, Asian, or Hispanic ancestry, with genetically-confirmed [36] European ancestry participants (the largest subgroup) serving as the reference group. Only measurements that improved model prediction of the specific lipid phenotype were retained in the final model.

Due to the high degree of correlation among the dietary fatty acid (DFA) measures, only those we had previously determined [29] to not be highly correlated with each other (pairwise correlation coefficient < 0.8) were included in this study. These DFA measures were: myristic acid (14:0), oleic acid (18:1), gadoleic acid (20:1), linolenic acid (18:3, a ω-3 DFA), arachidonic acid (20:4, an ω-6 DFA), and eicosapentaenoic acid (20:5, an ω-3 DFA).

Alcohol has previously been reported to increase HDL-C and its associated proteins; however, heavy alcohol use has also been reported to have the opposite effect [37]. Therefore, we created five groups of alcohol intake: 0 g/day, 0–12 g/day, 12–24 g/day, 24–60 g/day, and >60 g/day, as previously described by Framingham Heart Study investigators using the same food frequency questionnaire [38, 39]. Alcohol intake was then treated as a dummy variable compared to the reference of non-drinkers for stepwise linear regression analyses.

## Results

Demographic, clinical, selected dietary intakes, and lipid values are presented in Table 1. Participants of European ancestry comprised the majority of the cohort (77.1%), while participants of Asian (12.6%), African (8.2%), and Hispanic (2.1%) comprised the remainder of the selected subset of the CLEAR study. Males accounted for approximately two-thirds (63.3%) of the population. Of the studied subset, 32.9% were taking statins, 17.6% were diabetic, and 10.3% were current smokers. The average age of all participants was 65.3 years. All lipid phenotypes (apoA1, HDL-C, HDL-2, and HDL-3) showed approximate standard distributions.

**Table 1:** **Baseline characteristics of CLEAR study participants (n = 1566)**

Baseline characteristics	CLEAR cohort (N = 1566)
Ethnicity, n (%)	
European ancestry, not Hispanic	1207 (77.1)
Hispanic ancestry	33 (2.1)
African ancestry	128 (8.2)
Asian/Pacific Islander ancestry	198 (12.6)
Gender, n (%)	
Female	575 (36.7)
Male	991 (63.3)
Age, mean ± SD, years	65.3 ± 9.55
Current smoker, n (%)	161 (10.3)
Diabetic, n (%)	275 (17.6)
Statin Use, n (%)	515 (32.9)
Alcohol Intake	
0 = 0 g/day, n (%)	641 (40.9)
1 = 0 – 12 g/day, n (%)	588 (37.5)
2 = 12 – 24 g/day, n (%)	178 (11.4)
3 = 24 – 60 g/day, n (%)	126 (8.1)
4 = >60 g/day, n (%)	33 (2.1)
Dietary Micronutrient Intake	
Ln(Vitamin C intake), mg, mean ± SD	5.43 ± 1.00
Ln(Folate intake), μg, mean ± SD	6.71 ± 0.71
Ln(Iron intake), mg, mean ± SD	14.6 ± 6.53
Ln(Magnesium intake), mg, mean ± SD	5.79 ± 0.44
Ln(Carbohydrate intake), g, mean ± SD	5.26 ± 0.46
Ln(Protein intake), g, mean ± SD	4.36 ± 0.41
Ln(Dietary fiber intake), g, mean ± SD	2.92 ± 0.49
Ln(Myristic acid (14:0) intake), g, mean ± SD	1.01 ± 0.36
Ln(Arachidonic acid (20:4) intake), g, mean ± SD	0.14 ± 0.075
Ln(Eicosapentaenoic acid (20:5) intake), g, mean ± SD	0.14 ± 0.13
Fat Intake Composition	
Animal-based fat (of total fat intake), mean ± SD, %	37.4 ± 21.1
Vegetable-based fat (of total fat intake), mean ± SD, %	32.5 ± 18.8
Plasma Lipid Measures	
ApoA1, mean ± SD, mg/dl	150.5 ± 28.8
HDL-C, mean ± SD, mg/dl	54.4 ± 17.2
HDL-2, mean ± SD, mg/dl	11.1 ± 6.75
HDL-3, mean ± SD, mg/dl	43.3 ± 11.6

In addition to the demographic and clinical variables included in the base model, numerous dietary intakes increased total HDL-C variance explained in a stepwise linear regression model (see Table 2). All alcohol intake levels were positively associated with HDL-C. In addition, magnesium, folate, and the saturated fat, myristic acid (14:0), were all positively and independently associated with HDL-C. Carbohydrate intake, iron, and % of fat derived from animal sources were each negatively additive for HDL-C.

**Table 2:** **Best-fit model from stepwise linear regression predicting HDL-C levels using dietary intake data**

	Coefficient ± SE	%HDL-C variation	***P***
*(Intercept)*	*33.8 ± 7.78*	*-*	*-*
Current age, years	0.15 ± 0.04	0.15%	0.00016
Male gender	−13.6 ± 0.81	17.8%	<2×10^−16^
Current smoker	−2.25 ± 1.25	0.64%	0.067
Diabetic	−8.06 ± 1.00	4.42%	1.12×10^−15^
Statin Use	2.36 ± 0.82	0.63%	0.0039
Hispanic ancestry	−1.25 ± 2.53	0.072%	0.62
African ancestry	9.18 ± 1.44	0.51%	2.60×10^−10^
Asian ancestry	2.65 ± 1.16	0.0031%	0.022
24-60 g alcohol/day	11.3 ± 1.42	1.79%	2.99×10^−15^
12-24 g alcohol/day	7.23 ± 1.23	0.89%	4.98×10^−9^
0-12 g alcohol/day	5.19 ± 0.83	1.76%	4.73×10^10^
>60 g alcohol/day	11.0 ± 2.57	1.02%	1.95×10^−5^
Ln(Magnesium intake), mg	4.79 ± 1.45	0.12%	0.0010
Ln(Carbohydrate intake), g	−7.98 ± 1.35	1.22%	5.22×10^−9^
Ln(Folate intake), μg	5.25 ± 1.56	0.24%	0.00081
Ln(Iron intake), mg	−0.17 ± 0.10	0.32%	0.042
Ln(Myristic acid (14:0) intake), g	6.47 ± 2.08	0.12%	0.0018
Animal fat, %	−0.10 ± 0.04	0.29%	0.004

Similar effects from dietary intakes were observed for HDL-2: all alcohol intakes, magnesium, folate, and myristic acid (14:0) were each positively and independently associated with HDL-2 levels, while carbohydrate and iron intakes were both negatively associated with HDL-2 (see Table 3). Unique to HDL-2, arachidonic acid (20:4, an ω-6 DFA) was also negatively associated with HDL-2, while eicosapentaenoic acid (20:5, a ω-3 DFA) was positively associated with HDL-2.

**Table 3:** **Best-fit model from stepwise linear regression predicting HDL-2 levels using dietary intake data**

	Coefficient ± SE	%HDL-2 variation	***P***
*(Intercept)*	*2.95 ± 3.14*	*-*	*-*
Current age, years	0.070 ± 0.016	0.023%	1.53×10^−5^
Male gender	−5.18 ± 0.33	16.9%	<2×10^−16^
Current smoker	−0.022 ± 0.50	0.18%	0.96
Diabetic	−2.42 ± 0.40	2.82%	2.61×10^−9^
Statin Use	1.11 ± 0.33	0.76%	0.00086
Hispanic ancestry	−0.95 ± 1.03	0.096%	0.36
African ancestry	2.93 ± 0.58	0.43%	5.69×10^−7^
Asian ancestry	−0.24 ± 0.47	0.11%	0.61
24-60 g alcohol/day	3.39 ± 0.58	1.09%	5.33×10^−9^
12-24 g alcohol/day	1.78 ± 0.34	0.87%	1.38×10^−7^
0-12 g alcohol/day	1.88 ± 0.49	0.93%	0.00017
>60 g alcohol/day	2.63 ± 1.04	0.43%	0.012
Ln(Eicosapentaenoic acid (20:5) intake), g	3.52 ± 1.23	0.41%	0.0045
Ln(Magnesium intake), mg	1.43 ± 0.61	0.028%	0.018
Ln(Arachidonic acid (20:4) intake), g	−7.27 ± 2.54	0.37%	0.0043
Ln(Carbohydrate intake), g	−2.62 ± 0.55	0.81%	2.13×10^−6^
Ln(Folate intake), μg	2.07 ± 0.63	0.30%	0.0010
Ln(Myristic acid (14:0) intake), g	1.18 ± 0.51	0.18%	0.035
Ln(Iron intake), mg	−0.067 ± 0.041	0.13%	0.041

In the dietary models explaining HDL-3 variance, all alcohol intakes, magnesium, folate, and myristic acid (14:0) were positively associated with HDL-3 levels (see Table 4). Similar to HDL-C and HDL-2, HDL-3 levels decreased with increasing carbohydrate and iron intakes. Unique to HDL-3, increasing protein intake was associated with decreased HDL-3 levels, while dietary fiber was associated with increased HDL-3 levels.

**Table 4:** **Best-fit model from stepwise linear regression predicting HDL-3 levels using dietary intake data**

	Coefficient ± SE	%HDL-3 variation	***P***
*(Intercept)*	*41.2 ± 6.44*	*-*	*-*
Current age, years	0.072 ± 0.27	0.24%	0.0089
Male gender	−8.29 ± 0.55	15.1%	<2×10^−16^
Current smoker	−2.03 ± 0.84	0.89%	0.015
Diabetic	−5.69 ± 0.68	4.64%	<2×10^−16^
Statin Use	1.22 ± 0.56	0.46%	0.029
Hispanic ancestry	−0.47 ± 1.73	0.048%	0.79
African ancestry	6.04 ± 0.98	0.46%	7.97×10^−10^
Asian ancestry	2.72 ± 0.79	0.075%	0.00056
24-60 g alcohol/day	8.06 ± 0.98	1.91%	3.13×10^−16^
12-24 g alcohol/day	5.31 ± 0.84	1.12%	2.95×10^−10^
0-12 g alcohol/day	3.51 ± 0.57	1.64%	6.73×10^−10^
>60 g alcohol/day	8.48 ± 1.76	1.26%	1.56×10^−6^
Ln(Magnesium intake), mg	2.98 ± 1.20	0.085%	0.013
Ln(Carbohydrate intake), g	−5.52 ± 0.95	1.18%	7.34×10^−9^
Ln(Folate intake), μg	3.18 ± 1.11	0.19%	0.0044
Ln(Iron intake), mg	−0.15 ± 0.066	0.29%	0.025
Ln(Myristic acid (14:0) intake), g	3.48 ± 1.01	0.13%	0.00032
Ln(Protein intake), g	−3.35 ± 1.19	0.31%	0.0033
Ln(Dietary fiber intake), g	2.54 ± 1.10	0.23%	0.049

Finally, stepwise linear regression models considering dietary covariates to explain apoA1 variance identified similar trends: all alcohol intakes, folate, and myristic acid (14:0) were each positively and additively associated, while carbohydrate and iron intakes were negatively associated with apoA1 levels (see Table 5). Unique to apoA1, vitamin C intake was both positively associated with apoA1 levels. In addition to the aforementioned micronutrient intakes, increasing the percentage of fat derived from animal sources was associated with decreased apoA1 levels.

**Table 5:** **Best-fit model from stepwise linear regression predicting apoA1 levels using dietary intake data**

	Coefficient ± SE	%apoA1 variation	***P***
*(Intercept)*	*140.4 ± 13.8*	*-*	-
Current age, years	0.23 ± 0.067	0.16%	0.00054
Male gender	−24.3 ± 1.38	18.8%	<2×10^−16^
Current smoker	−4.24 ± 2.08	0.52%	0.041
Diabetic	−13.59 ± 1.67	3.77%	1.03×10^−15^
Statin Use	0.34 ± 1.38	0.89%	0.81
Hispanic ancestry	1.87 ± 4.28	0.0096%	0.66
African ancestry	13.9 ± 2.41	0.39%	8.67×10^−9^
Asian ancestry	6.53 ± 1.96	0.019%	0.00088
24-60 g alcohol/day	19.6 ± 2.39	1.72%	4.87×10^−16^
12-24 g alcohol/day	12.9 ± 2.07	0.92%	5.46×10^−10^
0-12 g alcohol/day	8.84 ± 1.39	1.66%	2.63×10^−10^
>60 g alcohol/day	21.5 ± 4.33	1.21%	7.95×10^−7^
Ln(Carbohydrate intake), g	−12.2 ± 2.44	0.31%	6.32×10^−7^
Ln(Myristic acid (14:0) intake), g	13.5 ± 3.54	0.64%	0.00014
Ln(Vitamin C intake), mg	1.21 ± 0.66	0.32%	0.048
Animal fat, %	−0.14 ± 0.068	0.24%	0.043
Ln(Folate intake), μg	6.69 ± 2.72	0.32%	0.014
Ln(Iron intake), mg	−0.42 ± 0.18	0.22%	0.017

A summary table of all the identified dietary micronutrient intakes and their respective associations to the studied lipid phenotypes (HDL-C, HDL-2, HDL-3, and apoA1) is presented in Table 6. All alcohol intakes, myristic acid (14:0), and folate were each positively and independently associated with all of the studied lipid phenotypes. Carbohydrate and iron intakes were both negatively and additively associated with all of the studied lipid phenotypes. Magnesium was positively associated with all HDL-specific (HDL-C, HDL-2, and HDL-3) measures. Percentage of fat from animal sources was negatively associated with both HDL-C and apoA1 levels. Unique to HDL-2, arachidonic acid (20:4, an ω-6 DFA) was negatively associated, while eicosapentaenoic acid (20:5, an ω-3 DFA) was positively associated with HDL-2 levels. For HDL-3 alone, dietary protein and dietary fiber intake were negatively associated. Finally, for apoA1 only, vitamin C was positively associated with apoA1 levels.

**Table 6:** **Summary of dietary intake associations with specific measures of the HDL-associated proteome**

Dietary intake	HDL-C	HDL-2	HDL-3	apoA1
0-12 g alcohol/day	++	++	++	++
12-24 g alcohol/day	++	++	++	+++
24-60 g alcohol/day	+++	++	++	+++
>60 g alcohol/day	+++	++	++	+++
Ln(Carbohydrate intake), g	---	--	--	---
Ln(Folate intake), μg	++	++	++	++
Ln(Iron intake), mg	-	-	-	-
Ln(Magnesium intake), mg	++	++	++	
Ln(Protein intake), g			--	
Ln(Dietary fiber intake), g			++	
Ln(Vitamin C intake), mg				++
Ln(Myristic acid (14:0) intake), g	++	++	++	+++
Ln(Oleic acid (18:1) intake), g				
Ln(Arachidonic acid (20:4) intake), g		--		
Ln(Eicosapentaenoic acid (20:5) intake), g		++		
Animal fat %	-			-

+ = between 0 and 1. ++ = between 1 and 10. +++ = greater than 10.

- = between 0 and −1. -- = between −1 and −10. --- = less than −10.

Sensitivity analyses by CAAD status and gender showed consistent effects of all the dietary covariates for all studied lipid phenotypes, suggesting that these factors were not affecting the relationship between the identified dietary covariates and their respective lipid measures.

## Discussion

Prior nutritional studies have generally focused on HDL-C alone. However, as measured HDL-C does not appear to be in the causative pathway for atherosclerotic disease [2–4], there is a growing recognition that any cardioprotective elements of HDL may be better captured by different measures of HDL [5]. In the current study, we have leveraged extensive dietary intake data within a large and well-characterized CAAD case–control cohort to identify the previously unreported predictors of HDL-2, HDL-3 and apoA1, and also validate numerous past associations with HDL-C. All of the HDL-related measures are highly correlated; thus, trends are seen in the dietary micronutrients and their associations with each of the lipid phenotypes. However, unique dietary associations were also elucidated for HDL-2, HDL-3, and apoA1 measures, which may reflect the differences among these HDL measures.

Low intakes of folate have previously been reported to be associated with higher incidence of CHD [40] and stroke [41] in prospective studies. One postulated mechanism for the observed cardioprotective properties of folate is through the folate-mediated lowering of homocysteine levels [42]. High levels of homocysteine are considered a modest and independent risk factor for heart disease and stroke [43]. In this study, we have what is, to the best of our knowledge, a novel finding: that dietary folate intake is also associated with favorable increases in HDL-related measures in humans. Prior epidemiologic evidence has positively linked plasma measures of folate with HDL-C [44] and apoA1 [45] levels. Although the increases in HDL-C, HDL-2, HDL-3, and apoA1 by folate intake are modest, they do represent a significant and consistent trend in our data and may represent a separate or complementary pathway through which folate mediates its cardioprotective effects.

All alcohol intake categories relative to non-drinkers were associated with an increase in all HDL-related measures. Gaziano *et al.* were one of the first to convincingly demonstrate that alcohol intake increased HDL-C, HDL-2, and HDL-3 levels, which they posited as a possible mechanism for decreased myocardial infarction among drinkers [46]. In addition, Gaziano *et al.* also reported that moderate (between 13.2 and 39.6 g/day) alcohol intake caused the greatest increase in HDL-2, while at heavy drinking (>39.6 g/day) HDL-2 levels decreased; HDL-C and HDL-3 continued to rise with increasing intake of alcohol [46]. We observed similar trends in our present work, as HDL-C and HDL-2 levels both decreased with heavy alcohol intake (>60 g/day); similarly, though HDL-3 and apoA1 increased at the highest levels of alcohol consumption, the gains were more modest in comparison to the subgroups with lower levels of alcohol intake. Interestingly, in a prospective study of 80,082 women, Rimm *et al.* reported that the cardioprotective effects of folate were most pronounced in women who consumed alcohol [40]. Though underpowered, we did not find evidence of an interaction between alcohol and folate intake on any measured HDL phenotype in our data (data not shown).

Both dietary carbohydrate and iron intakes were negatively associated with all measured HDL phenotypes. Dietary carbohydrates have previously been reported to decrease HDL-C levels [47, 48]. However, many of the studies were small (n = 10 and n = 8, respectively for the cited works), did not measure HDL-2 or HDL-3, and were short-term dietary intervention studies. In the work presented here, we have identified a strong, consistent, and negative association between carbohydrate intake and all HDL-measures (HDL-C, HDL-2, HDL-3, and apoA1) that reflects dietary intake over the past year, rather than a period of days. Similarly, high body stores of iron and dietary iron intake have been associated with increased risk of CHD in 1,931 Finnish men [49]. In addition, ferritin levels were negatively correlated with HDL-2 levels in that cohort of men [49]. Our findings are consistent with past reports of a negative association between HDL-2 and iron levels, and also show a consistent and negative effect of dietary iron on all HDL-related measures.

Dietary magnesium intake was positively associated with HDL-C, HDL-2, and HDL-3 levels, but not associated with apoA1 in our data. Singh *et al*. first reported from a randomized clinical trial of 430 patients that a magnesium-rich diet increased HDL-C levels at 12 weeks of follow-up [50]. Although the mechanism through which magnesium influences HDL-C levels is yet unknown, the positive association of dietary magnesium intake and HDL-C has been validated in a subsequent prospective study of 4,637 participants [51]. Here we expand on these findings and report that magnesium also increases levels of HDL-2 and HDL-3, but not apoA1. Further work will be needed to determine why magnesium affects levels of HDL, but not its closely related protein, apoA1.

Prior work on DFAs and lipid profiles have largely focused on the ω-3 polyunsaturated fatty acids commonly found in fish [23, 52, 53]. In this work we have found that the most significant and consistent DFA intake impacting HDL-related measures was that of the saturated fat, myristic acid (14:0). Saturated fats, including myristic acid, have been previously been reported to increase HDL-C [54]; however, they also have been reported to simultaneously increase low-density lipoprotein cholesterol levels [54, 55]. We did observe potentially protective effects of an ω-3 fatty acid, eicosapentaenoic acid (20:5), through its positive association with HDL-2 levels. We also noted a negative association of HDL-2 and arachidonic acid (20:4, ω-6). Arachidonic acid is most commonly found in meat and dairy.

Several limitations of this study should be considered. First, this cohort was composed of a majority of European Ancestry and participants selected for the presence or absence of CAAD, limiting the generalizability of our findings. Second, trans-fatty acids could not be included in analyses due to a high proportion of missing data. Unsaturated trans fatty acids have previously been associated with higher risk of CHD [56]; therefore, future analyses should consider the effect of trans fatty acids on the HDL proteome. Third, the dietary data analyzed in this study was limited to what was measured by the Harvard Standardized FFQ. As a result, it was not possible to evaluate other micronutrients, such as the flavonoids (e.g., quercetin), which have also been linked to favorable alterations to cardiovascular and neurodegenerative risk factors [57, 58]. Strengths of this study include the large sample size, extensive lipid phenotyping, and detailed demographic, clinical, and pharmacologic information coupled with a well-validated food frequency questionnaire.

In conclusion, we present the first known report of dietary folate intake affecting HDL-related phenotypes (HDL-C, HDL-2, HDL-3, and apoA1) in humans, thereby offering another potential mechanism independent of homocysteine through which folate mediates its cardioprotective effects. This study is also the first to report and validate numerous associations of dietary micronutrients with the more specific measures of HDL: HDL-2 and HDL-3. Given the recent failures of HDL-C to demonstrate cardioprotection, this work improves knowledge of how dietary factors influence the more specific measures of HDL (HDL-2, HDL-3, and apoA1). Future work should investigate the biologic mechanisms and pathways through which these micronutrients affect HDL and how specific micronutrients, such as vitamin C, might affect apoA1 but not any of the other measures of HDL.
